# *Bacteroides xylanisolvens* possesses a potent anti-hyperuricemia effect in goslings fed on a high-protein diet

**DOI:** 10.3389/fmicb.2023.1173856

**Published:** 2023-06-30

**Authors:** Ning Song, Mingze Wang, Guangxu Zhong, Kunpeng Zhu, Pengju Chen, Naisheng Zhang, Xiaobo Liu, Wenlong Zhang

**Affiliations:** ^1^Department of Clinical Veterinary Medicine, College of Veterinary Medicine, Jilin University, Changchun, China; ^2^Henan Institute of Modern Chinese Veterinary Medicine, Zhengzhou, Henan, China; ^3^Shandong Xindehui Biotechnology Company Ltd., Yuncheng, Shandong, China; ^4^School of Life Science, Jilin University, Changchun, China

**Keywords:** intestinal flora, hyperuricemia, uric acid, *Bacteroides xylanisolvens*, goslings

## Abstract

**Introduction:**

Hyperuricemia is widespread in humans and birds which is a necessary physiological factor leading to gout. Studies have shown an inextricable relationship between gut microbiota and hyperuricemia. This study explored the association between intestinal flora and hyperuricemia in Goslings.

**Methods and results:**

The hyperuricemia model was established in gosling by a high protein diet (HPD). 16S rDNA sequencing showed that the cecal microbiota differed significantly between the HPD and control groups (fed with the normal protein). The abundance of *Firmicutes* was higher in the HPD group, while the *Bacteroidetes* were lower than in controls. To investigate the role of intestinal flora in hyperuricemia, the cecum microbiotas from the HPD group and the control group were transplanted to the newly born goslings by gavage. The serum uric acid levels of the goslings that transplanted the cecal microbiota of the HPD group were significantly higher than the goslings that transplanted the cecal microbiota of the controls. Furthermore, the transplantation of cecal microbiota also affects the production and excretion of uric acid in goslings. Then we identify the gut bacterium *Bacteroides xylanisolvens* as an effective anti-hyperuricemia in the Goslings. *B. xylanisolvens* reduces serum uric acid concentrations in hyperuricemia in the Goslings' model, and it can up-regulation ABCG2 mRNA expression in the kidney and down-regulation XDH mRNA expression in the liver.

**Discussion:**

The intestinal flora acts as a novel target for the therapeutic approach to hyperuricemia and gout, suggest *Bacteroides xylanisolvens* is a possible route to therapy for hyperuricemia and gout in goslings.

## Introduction

Among mammals and birds, gout is the most common metabolic disease, which is inflammatory arthritis associated with hyperuricemia. A concentration-dependent link between serum uric acid and the risk of developing gout has been discovered (Campion et al., 1987). Individuals with hyperuricemia have a serum uric acid level higher than 360 μmol/L for women or 420 μmol/L for men (Lin et al., 2000). Hyperuricemia and gout are driven by disruption in purine metabolism homeostasis, including genetic and environmental factors. Most exogenous purines are formed in the liver, the intestinal tract, and the endothelium of veins. Adenine and guanine, which are derived from damaged and dead cells, are endogenous purines. Then, these two kinds of purines are degraded into uric acid (UA) (El Ridi and Tallima, 2017). Serum uric acid (SUA) is also influenced by high protein diets or other dietary factors (such as a high purine diet) that increase the risk of gout incidents (Choi et al., 2004; Choi, 2010).

The excretion of urate and the concentration of serum urate is physiologically related. In avian specimens, ~10% of the excretion passes through the intestinal tract, and 90% passes through the kidneys, significantly increasing serum urate levels. In poultry, urate oxidase and glutamine synthetase were lacking. Therefore ammonia was hardly excreted (Ma et al., 2021). Avian gout develops because of disruption of blood chemistry caused by high serum uric acid (SUA). It is very common for poultry, especially goslings, to suffer from hyperuricemia and gout. After hatching, goslings usually reach this stage between 7 and 15 days. The absence of the urate oxidase enzyme in young goslings increases their risk of gout because poorly-soluble uric acid (UA) is transformed into water-soluble allantoin, which elevates serum uric acid levels. In 2016, there was a large-scale outbreak of gout in the goose industry in China, which resulted in a 50% increase in geese mortality (Zhang et al., 2018).

Research on urate transporters in the kidney is more advanced than that of the gut system (Ichida et al., 2012). There are also several unidentified urate transporters. Further, ABCG2 has also played a crucial role in urate excretion in the digestive tract (Xu et al., 2016). The impact of the gut microbiota on the host's health and sickness is also becoming more understood. The intestinal microbiota of gout patients has been found to be unbalanced, which may be an indicator of the condition (Guo et al., 2016). Probiotics have been established to have the ability to degrade UA (Wu et al., 2021). In a prior investigation, normal mice were given transplants of HUA-containing mouse feces, and the normal mice's SUA concentration increased (Liu et al., 2020). The metabolism of purine and uric acid was also carried out by the gut microbiota (Guo et al., 2016).

Poultry, especially goslings, can suffer from hyperuricemia and gout (Ma et al., 2021). In particular, goslings suffer from gout at an early age in poultry farming (Xi et al., 2019). However, studies on the connection between gut flora with hyperuricemia and gout in the goose are currently scarce. This study intended to explore the relationship between hyperuricemia and gut microbiota in goslings. To the best of our knowledge, the study's most notable discovery is that the gut microbiota had a significant impact on the emergence of hyperuricemia and the excretion of uric acid. It indicated that the intestinal flora correlates with the pathogenesis of hyperuricemia and serves as a novel target for the therapeutic approach to hyperuricemia and gout. In that context, we found that Bacteroides may play a profound role in preventing Hyperuricemia and gout in goslings.

## Materials and methods

### Ethics statement

The Institutional Animal Care and Use Committee at Jilin University approved the entire protocol, including the Sanhua geese autopsy and sample collection (20170318).

### Animals and experimental design

Sanhua geese were purchased from Longde Biotechnology Co., LTD (Changchun, Jilin, China). All the newly born goslings (male) Sanhua geese were perched in a net bed for the entire experiment. All geese had a heat lamp for the first week to maintain the temperature. A final temperature of 26°C was reached after the first week's temperature of 32 ± 1°C was lowered by 2.5 ± 0.5°C each week. Over the whole 25-day experimental period, the geese were allowed a daily ration and water.

The first experiment used 1-day-old male Sanhua geese, which were fed experimentally for 25 days. They were divided into two groups at random. As presented in Table 1, the control group (normal protein diet) had a basal diet containing 20.75% crude protein; HPD (high-protein diet) consisted of 36.59% crude protein.

**Table 1:** The basic ingredients of feed.

**Item**	**Unit**	**Normal protein feed**	**High protein feed**
Water content	%	10.1	9.6
Coarse ash	%	4.6	11.4
Crude protein	%	20.75	36.59
Crude fiber	%	3.6	5.2
Calcium	%	0.646	1.94
Phosphorus	%	0.57	1.09
Water soluble chloride	%	0.30	0.80
Methionine	g/100 g	1.84	4.42
Lipid	g/100 g	2.6	1.8
Carbohydrate	g/100 g	60.4	41.0
Purine	mg/kg	256	344
Adenine	mg/kg	115	194
Guanine	mg/kg	40.4	77.4
Xanthine	mg/kg	99.8	73.5
Digestive energy	kcal	34,011.9	33,058.9

Vitamin premix provided the following per kilogram of diet: vitamin A, 40,000,000 IU; vitamin D 3, 10,000,000 IU; vitamin E, 100,000 mg; vitamin K 3, 20,000 mg; vitamin B 1, 10,000 mg; vitamin B 2, 30,000 mg; vitamin B 6, 20,000 mg; vitamin B 12, 12,100 mg; biotin, 500 g; pantothenic acid, 60,000 mg; folic acid, 5,000 mg; nicotinic acid, 200,000 mg; ethoxyquin, 500 mg. The mineral premix provided the following per kg of diet: Cu, 8–12 g; Fe, 100–110 g; Mn, 120–130 g; Co, 0.3–0.5 g; Se, 0.3–0.5 g; I, 0.7–0.9 g; moisture ≤ 3%.

**Figure 1:**
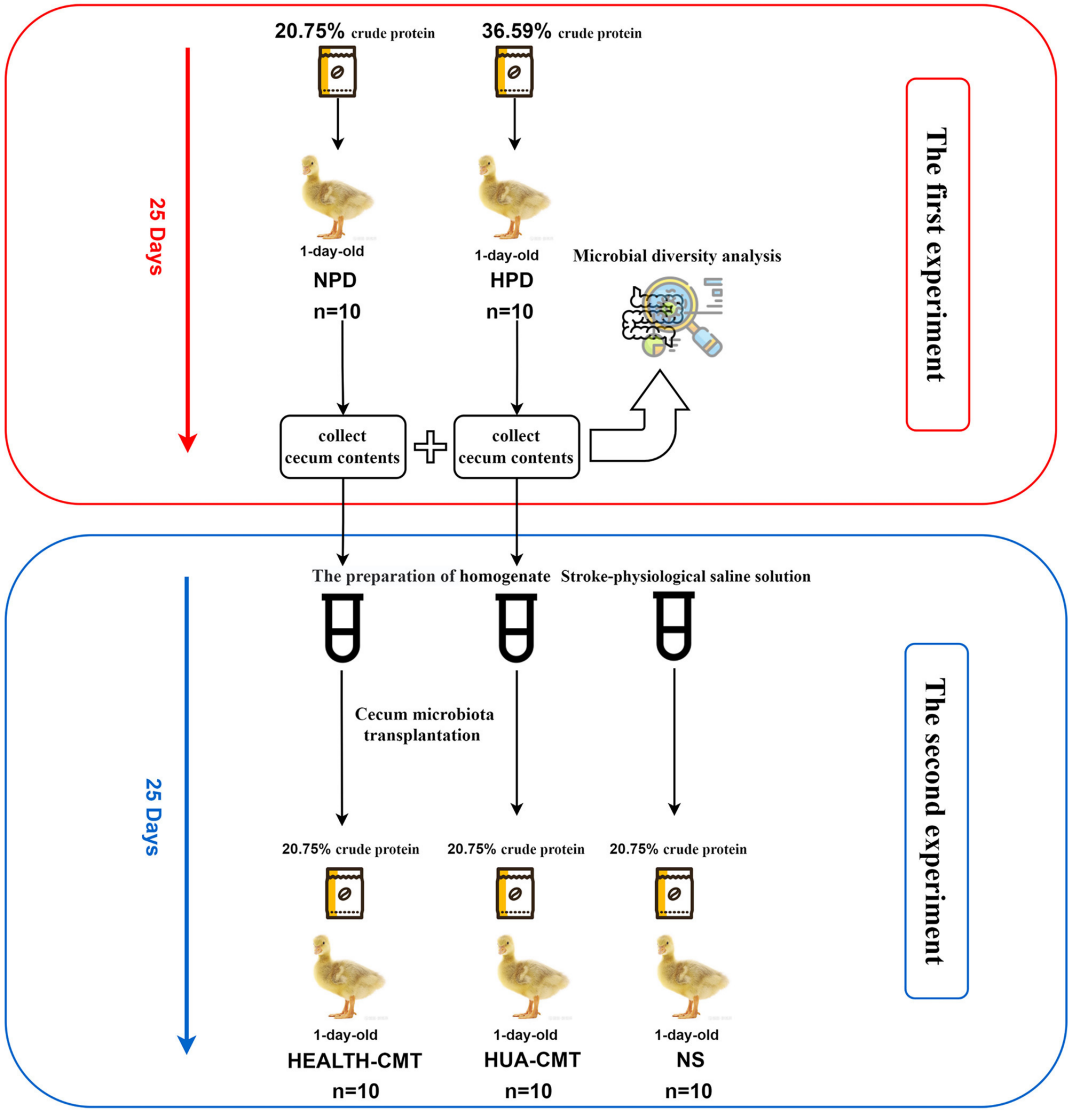
The experimental process.

The second experiment used 1-day-old male Sanhua geese, which were fed experimentally on a normal protein diet for 25 days. They were randomly assigned to the NS group (treated with sterile normal stroke-physiological saline solution, *n* = 10), HEALTH-CMT group (transplanted with cecal microbiota of the control group, *n* = 10), HUA-CMT group (transplanted with cecal microbiota of the HPD group, *n* = 10). These geese were fed a normal protein diet (containing 20.75% crude protein). The first and second experiment process is shown in Figure 1.

The third experiment used 1-day-old male Sanhua geese, which were fed experimentally for 25 days. They were divided into five groups at random. They were randomly assigned to the CONTROL group (*n* = 10), HPD group (treated with a high-protein diet, *n* = 10), CONTROL + *Bacteroides xylanisolvens* group (treated with a normal protein diet, and *Bacteroides xylanisolvens* given 10^10^ CFU/mL by gavage, *n* = 10), HPD + *Bacteroides xylanisolvens* group (treated with a high-protein diet and *Bacteroides xylanisolvens* given 10^10^ CFU/mL by gavage, *n* = 10), and HPD + Allopurinol group [treated with a high-protein diet and Allopurinol (J&K Scientific, Beijing, China) given 10 mg/kg by gavage, *n* = 10].

### Cecum microbiota transplantation

The previously described procedure of transplanting feces microbiota was used (Pang et al., 2007; Hamilton et al., 2012). Cecum contents were collected from 18 goslings (Control = 8, HPD = 10). Cecum contents of every group were, respectively, mixed with sterile normal stroke-physiological saline solution (1 g/2 mL of NS) and homogenized in a commercial blender immediately. The homogenate was centrifuged (1,000 × g, 15 m, 4°C), and the supernatant of every group was directly dispensed to 25 cryotubes, which contained 10% glycerol (v/v), and stored at −80°C until used. The frozen preparation was diluted 20-fold in sterile normal stroke-physiological saline solution for transplantation. A total of 1 mL of the supernatant was administered to newly born goslings by oral gavage for 25 consecutive days. The goslings of the control group were issued an equal volume of sterile normal stroke-physiological saline solution (NS group).

### Microbial diversity analysis

#### Cecum contents collection

Following the collection of blood samples, the cecum contents of 18 goslings (CONTROL = 8, HPD = 10) were collected in 5 mL sterile internally threaded cryogenic vials and immediately stored in liquid nitrogen before being stored at −80°C.

#### DNA extraction and 16s rDNA amplicon sequencing

Using the CTAB or SDS method, the genomic DNA of the cecal microbiota was extracted, and the purity and quantity of the DNA were then determined by agarose gel electrophoresis. The right amount of sample DNA was added to a centrifuge tube and diluted with sterile water to 1 ng/μl. To amplify DNA accurately and efficiently, we used specific primers with Barcode and Phusion^®^ High-Fidelity PCR Master Mix with GC Buffer from New England Biolabs. This process involved using diluted genomic DNA as a template and selecting the sequencing region for PCR. The ratios of 260/280 nm and 260/230 nm were used to evaluate the quantity and quality of DNA. The 16S v3-v4 region of bacteria was subjected to PCR using a primer pair 515F and 806R (5′-GTGYCAGCMGCCGGTAA-3′ and 5′-GGACTACNVGGGTWTCTAA-3′) (Caporaso et al., 2012). The qualified PCR products were purified with magnetic beads and quantified with enzyme labeling. The samples were then mixed in equal portions according to the concentration of the PCR products before being used with 2% agarose gel after thorough mixing. The PCR products were detected by electrophoresis on agarose gel with a concentration of 2%. The target bands were recovered using the gel recovery kit provided by Qiagen, China after the PCR products were identified by glycogen electrophoresis. The library was made using the TruSeq^®^ DNA PCR-Free Sample Preparation Kit. The built-in library was quantified using Q-PCR and qubit. After library qualification, on-machine sequencing was performed using NovaSeq6000.

#### Sequencing data processing

Each sample data was split from offline data according to barcode sequence and PCR amplification primer sequence. We cut the barcode and primer sequence to analyze each sample's reads and used FLASH (V1.2.7, http://ccb.jhu.edu/software/FLASH/) (Magoc and Salzberg, 2011) splicing. The splicing sequence obtained was the original tag data (Raw Tags), according to Qiime's tag quality control process (V1.9.1, http://qiime.org/scripts/split_libraries_fastq.html) (Caporaso et al., 2010). The Raw Tags were then filtered rigorously to obtain high-quality tag data (Clean Tags) (Bokulich et al., 2013). The initial base with poor quality was identified and removed if its length was less than the set default of 3. The resulting tag data set was then filtered further, dropping any tags that were <75% of the set length. The tags were then aligned with the species annotation database (https://github.com/torognes/vsearch/) to detect any chimera sequence (Asshauer et al., 2015) and remove them to get the final practical data (Effective Tags). The PCR-free library was constructed based on the Illumina Nova sequencing platform, and then paired-end (Paired-End) sequencing was performed. Through the splicing of the reads, an average of 85,746 tags were measured per sample, and an average of 79,488 valid data were obtained after quality control. The amount of accurate data for quality control reached 53,117, and the effective quality control rate reached 61.86%. The sequences were clustered into OTUs (Operational Taxonomic Units) with 97% identity (Identity), and a total of 2,452 OTUs were obtained. The OTU sequences were then species-annotated with the Silva138 database. In the annotated results, a total of 755 (30.79%) OTUs were annotated to the genus level. The predicted meta-genomes and gut microbiota function were inferred using Tax4FUN (Asshauer et al., 2015). Based on the Kyoto Encyclopedia of Genes and Genomes (KEGG) database, these predictions were made in advance for the annotation of genes. Distinct gene categories were chosen based on the gene categories' significant distinctions at level 3. The PCoA analysis was undertaken using the WGCNA package, stat packages, and ggplot2 package in R software (Version 2.15.3) (Prentice and Eddy, 1987).

### Isolation of the bacterial strain and 16s rDNA gene sequencing and phylogenetic analysis

According to the 16S rDNA sequencing results, the contents were extracted aseptically from the cecum of healthy goslings and then cultured in a specific screening medium at 37°C under anaerobiosis (Table 2). When single colonies grew in the medium, it was selected and cultured in BHI liquid medium (purchased from Qingdao Hope Bio-Technology Co., LTD). To confirm *Bacteroides xylanisolvens*, 2 × 10^10^ cells/ml of bacteria were centrifuged at 12,000 × g for 10 m. Then the supernatant was discarded to obtain a precipitate. The DNA was then extracted using the TIAN-amp DNAMinikit. The 16S rDNA was amplified as described by Wooden et al. (1993). Using the BLAST engine of the National Center for Biotechnology Information (NCBI), we identified potential strains for species identification and submitted their nucleotides to GenBank. Then, to confirm it, we created a pair of specific primers, the forward primer being 5′-CGCTTTCAGCTCCTGAGA-3′ and the reverse primer being 5′-ATGCCCACGAGTGATTGACC-3′. The primers underwent a 35-cycle PCR program that included predenaturation at 94°C for 3 m, denaturation at 94°C for 30 s, annealing at 60°C for 30 s, extension at 72°C for 30 s, and an extension at 72°C for 5 m. Approximately 1 μl of each primer, 1 μl of DNA template, 12.5 μl of Taq, and 9.5 μl of sterile water made up the reaction. When it was confirmed that the isolated strain was Bacteroidetes, then its growth curve was measured.

**Table 2:** Bacteroidetes specific medium component.

**Item**	**Unit**	**Weight**
Peptone	g/L	10.0
Yeast extract fermentation	g/L	2.0
Casein tryptone	g/L	10.0
NaCl	g/L	5.0
Glucose	g/L	1.0
NaHSO3	g/L	0.1
Agar	g/L	15.0
Fetal bovine serum	ml	50
Hemin	g/L	0.01
Kanamycin	g/L	0.1
Vancomycin	g/L	0.0075

### Preparation of *Bacteroides xylanisolvens* gavage

*Bacteroides xylanisolvens* were cultured in a BHI liquid medium at 37°C under anaerobiosis. When the number of well-cultivated *Bacteroides xylanisolvens* reached 2 × 10^10^ cells/ml, 25% of glycerol (v/v) was added and stored at −80°C until used. Before starting the gavage, *Bacteroides xylanisolvens* were harvested through centrifugation (4,200 g for 10 m), washed in sterile normal stroke-physiological saline solution, and re-suspended in sterile normal stroke-physiological saline solution (NS). Then 1 ml of *Bacteroides xylanisolvens* solution was gavaged per gosling.

### Quantitative real-time PCR

Quantitative RT-PCR was used to isolate and examine total RNA. Reverse transcription was carried out using the PrimerScript RT reagent kit with gDNA Eraser (Transgen, China) according to the manufacturer's instructions. The primers for the upstream and downstream were created using DNAMAN software. Table 3 displays the sequence of the primers. On the real-time fluorescent quantitative PCR instrument Applied Biosystems 7500 (Thermo Fisher, USA), fluorescent quantitative PCR was carried out using the SYBR Green Quantitative PCR kit (Takara, Japan). The 2^−Δ*ΔCT*^ method was used to determine the target gene's relative expression.

**Table 3:** Primer sequences for RT-PCR.

**Gene**	**Product length (bp)**	**Forward primer 5^′^-3^′^**	**Reversed primer 5^′^-3^′^**
β-actin	158	CATGGGGCAGAAAGACAGCTA	CGGGGGCCACACGTAATTCA
ADA	125	CGCTGTCGCTTACCGAGTTT	GACGACGCCTTCCTTCGCTTT
ABCG2	126	ACTCCATATTCCGGCTGTTTGAC	TGTTGTAAGGCTCGCACTCGTAG
SLC2A9	242	GCATTGGCTTCCTTACCTCA	AGCAGATGGCAGCAAACACT
XDH	202	GAACAAACCTGAACCCAACAT	TCTTCCTCCACAACAACTCCC

ADA, Adenosine deaminase; ABCG2, ATP-binding cassette sub-family G member 2; SLC2A9, solute carrier family 2 member 9; XDH, xanthinedehydrogenase.

### Serum biochemistry analyses

Serum uric acid was detected using a fully automatic biochemical analyzer (SUA) (Catalyst One, Inc., IDEXX, USA). Serum creatinine (Cr), blood urea nitrogen (BUN), and Lipopolysaccharide (LPS) were measured using enzymatic kits from Nanjing Jiancheng Bioengineering Institute, Nanjing, China.

### Kidney and histology analysis

The kidney was placed in 10% buffered formalin, and 5-μm paraffin-embedded sections were stained with hematoxylin and eosin (H&E). Finally, pathological changes were examined and graded as described previously using a microscope (Olympus, Japan) (Cao et al., 2011).

### Statistical analysis

All values were expressed as the means ± SEM. Raw data were subjected to one-way ANOVA to evaluate statistical significance between at least three groups, and pairwise comparison was conducted using Student's *t*-test. The results were considered statistically significant at *p* < 0.05. Shannon, Simpson, Chao1, and ACE indices in our samples were calculated with QIIME (Version 1.7.0) and displayed with R software (Version 2.15.3). Beta diversity on weighted unifrac was calculated by QIIME software (Version 1.9.1). PCoA analysis was displayed by the WGCNA package, stat packages, and ggplot2 package in R software (Version 2.15.3) (Prentice and Eddy, 1987). A distance matrix of weighted unifrac from the samples obtained before was transformed to a new set of orthogonal axes, by which the maximum variation factor was demonstrated by the first principal coordinate; the second maximum variation factor was determined by the second principal coordinate. To study the significance of the differences in community structure between groups, the adonis and anosim functions in the QIIME2 software were used. The LEfSe software (Version 1.0) was used for the LEfSe analysis to identify the biomarkers (Edgar, 2004; Segata et al., 2011). To determine the significantly different species at each taxonomic level (Phylum, Class, Order, Family, Genus, Species) (Edgar, 2004; Wang et al., 2007), the R software (Version 3.5.3) was used to undertake MetaStat and *t*-test analysis. Further, to study the functions of the communities in the samples and determine their different functions in the different groups, the Tax4fun software was used for functional annotation analysis.

## Results

### HPD causes hyperuricemia in goslings

**Figure 2:**
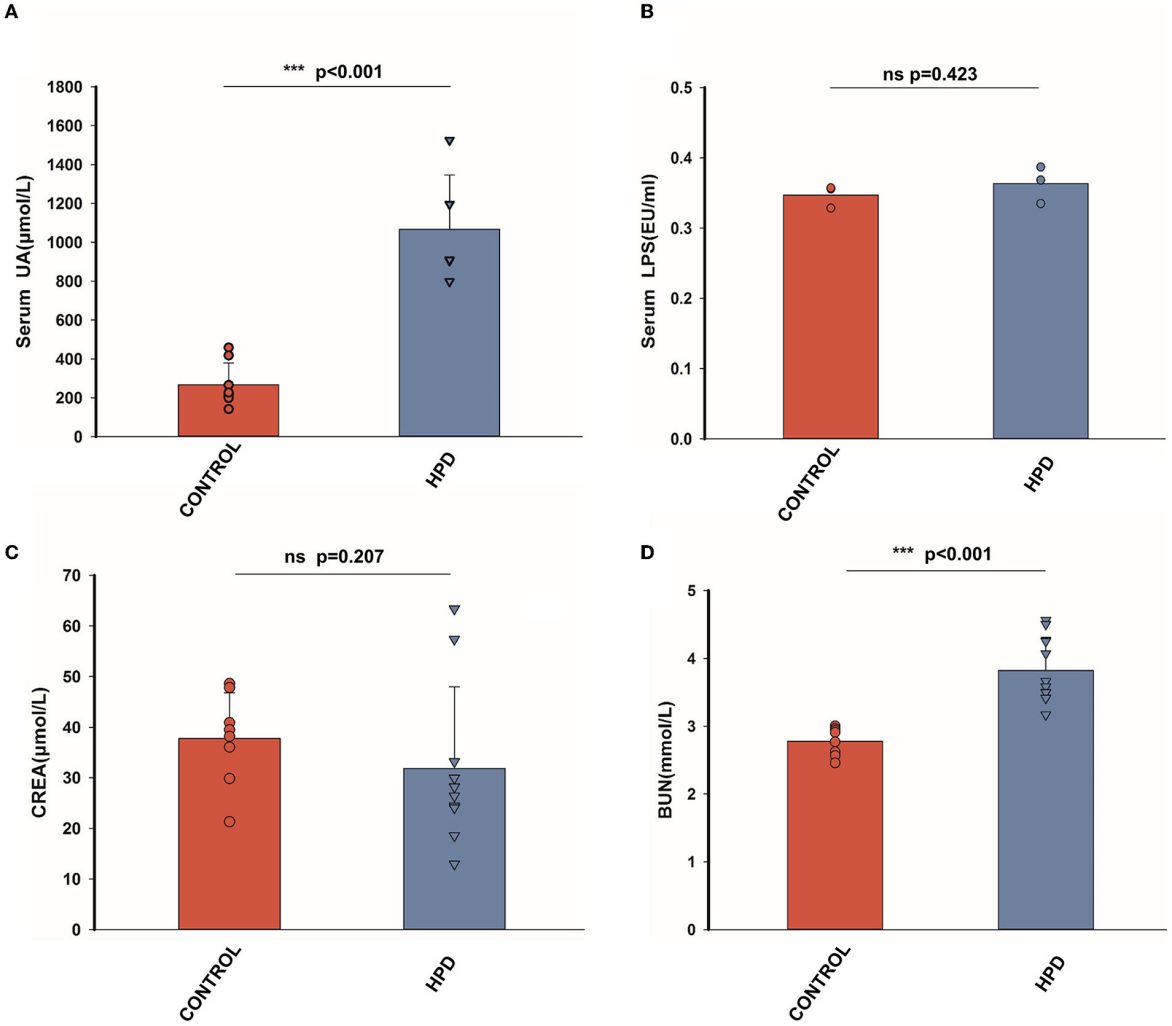
The effect of HPD on biochemical parameters in goslings. **(A)** The serum uric acid levels of the goslings of the three groups. **(B)** The serum LPS levels of the goslings of the three groups. **(C)** The serum creatinine levels of the goslings of the two groups. **(D)** The blood urea nitrogen levels of the goslings of the three groups. Values are presented as the mean ± SEM. Differences were assessed by One Way ANOVA by the Holm-Sidak method and denoted as follows: *p < 0.05, **p < 0.01, and ***p < 0.001.

After a 25-day experiment with HPD or a normal protein diet, serum uric acid levels of goslings were tested. Serum uric acid levels were higher in HPD groups than in the controls (Figure 2A). All the goslings from the HPD groups were hyperuricemia (uric acid level > 420 μmol/L). Although the levels of serum LPS and serum creatinine (CREA) were comparable between the HPD groups and the controls (Figures 2B, C), the blood urea nitrogen (BUN) concentration of the HPD group was higher than the controls (Figure 2D). These findings demonstrated that gosling hyperuricemia could result from a high-protein diet.

### Significant changes in intestinal flora occur in goslings with hyperuricemia

**Figure 3:**
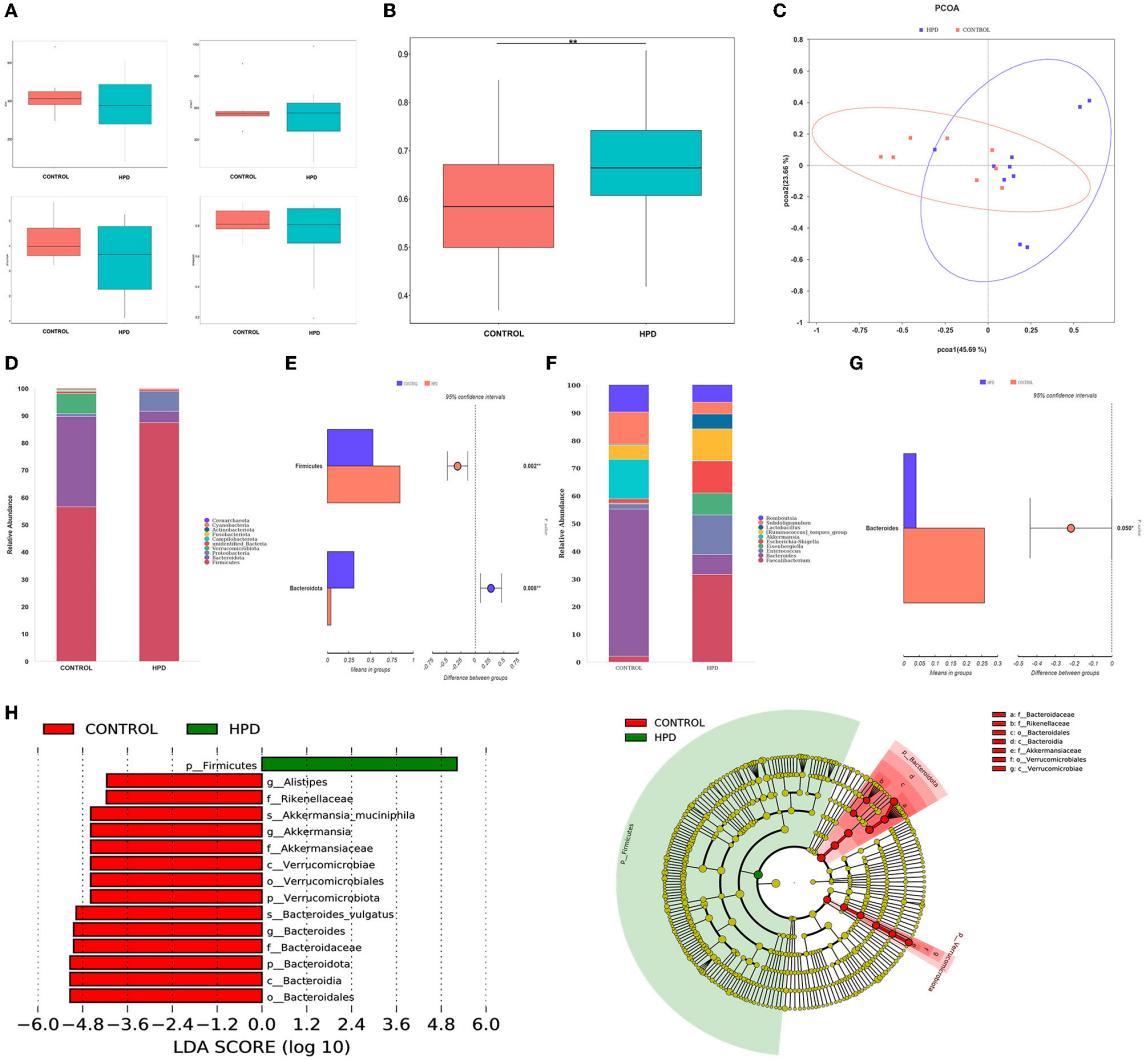
Changes of cecal microflora in gosling after hyperuricemia. **(A)** Alpha diversity index (the ACE, Chao1, Shannon, and Simpson indices). **(B)** β-diversity (unweighted Wilcox). **(C)** PCoA analysis. **(D)** The overall composition of the microbiota and average relative abundance in the two groups at the phylum. **(E)** The significant difference in microbiota of each group at phylum. **(F)** The microbiota's overall composition and average relative abundance in the two groups at the genus. **(G)** The significant difference in microbiota of each group at phylum. **(H)** LEfSe analysis. Differences were assessed by t-test and denoted as follows: ^**^p < 0.01.

Species richness, evenness, and rarity are the critical components of biodiversity and are usually measured by Shannon, Simpson, Chao1, and ACE indices. In our results, the ACE, Chao1, Shannon, and Simpson indices were not significantly different (Figure 3A). However, the beta diversity results showed that the HPD group was quite different (*p* < 0.05) from the control group (Figure 3B). Furthermore, principal-coordinates analysis (PCoA) results showed that phylogenetic community structures were markedly different between HPD and the control group (Figure 3C). It suggested that gut microbiota plays an essential role in hyperuricemia. The relative abundance at each group's top 10 phylum levels was analyzed, as shown in Figure 3D. In the HPD group, the abundance of Firmicutes significantly increased, and a significant reduction in the Bacteroidetes (Figure 3E) was observed. The relative abundance at each group's top 10 genus levels was analyzed, as shown in Figure 3F. Comparing the two groups, the abundance of Bacteroides was significantly different from each other, and in the HPD group, a significant reduction in the Bacteroides (Figure 3G) was noted. We examined the abundance of the two types of microflorae to further analyze the primary biomarker of the cecal microbiota that affects hyperuricemia. Using LEfSe analysis, we could distinguish between the various major microbiota. It was found that the abundance of *Bacteroidetes* increased in the control group (Figure 3H). These results showed that significant changes in intestinal flora occurred in goslings with hyperuricemia.

### Predicts the functional capabilities of microbial communities

**Figure 4:**
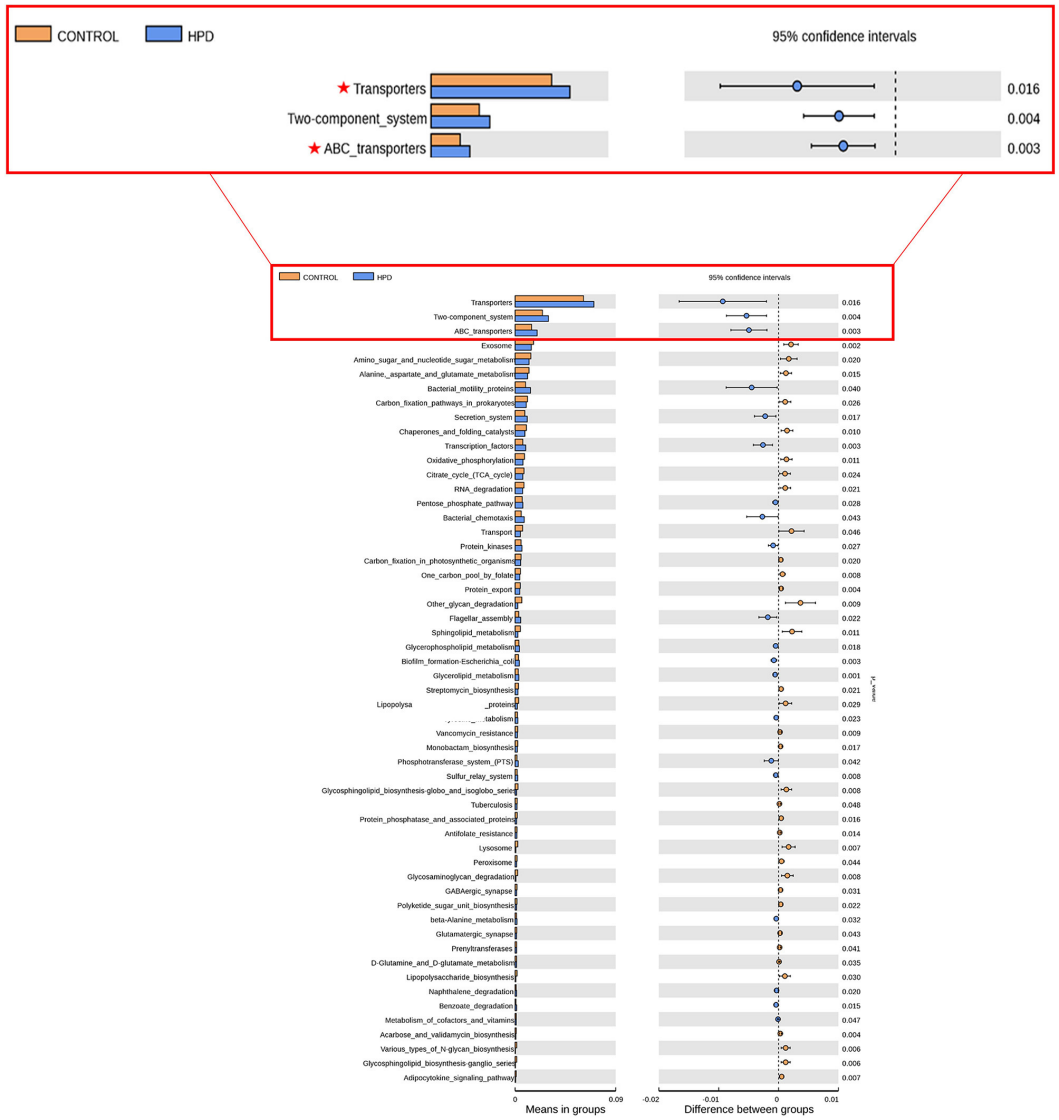
Predictions of the functional capabilities of microbial communities. Significant differences in gene categories between the HPD group and the CONTROL group. Distinct gene categories were selected based on significant differences in gene categories at level 3 (*t*-test, *p* < 0.05).

Upon further investigation, compared with the control and HPD groups, the KEGG analysis of bacterial community functions revealed that transporters and ABC_transpoters differed significantly (Figure 4).

### Cecal microbiota transplant from HPD groups to the newly born goslings elevates uric acid levels

**Figure 5:**
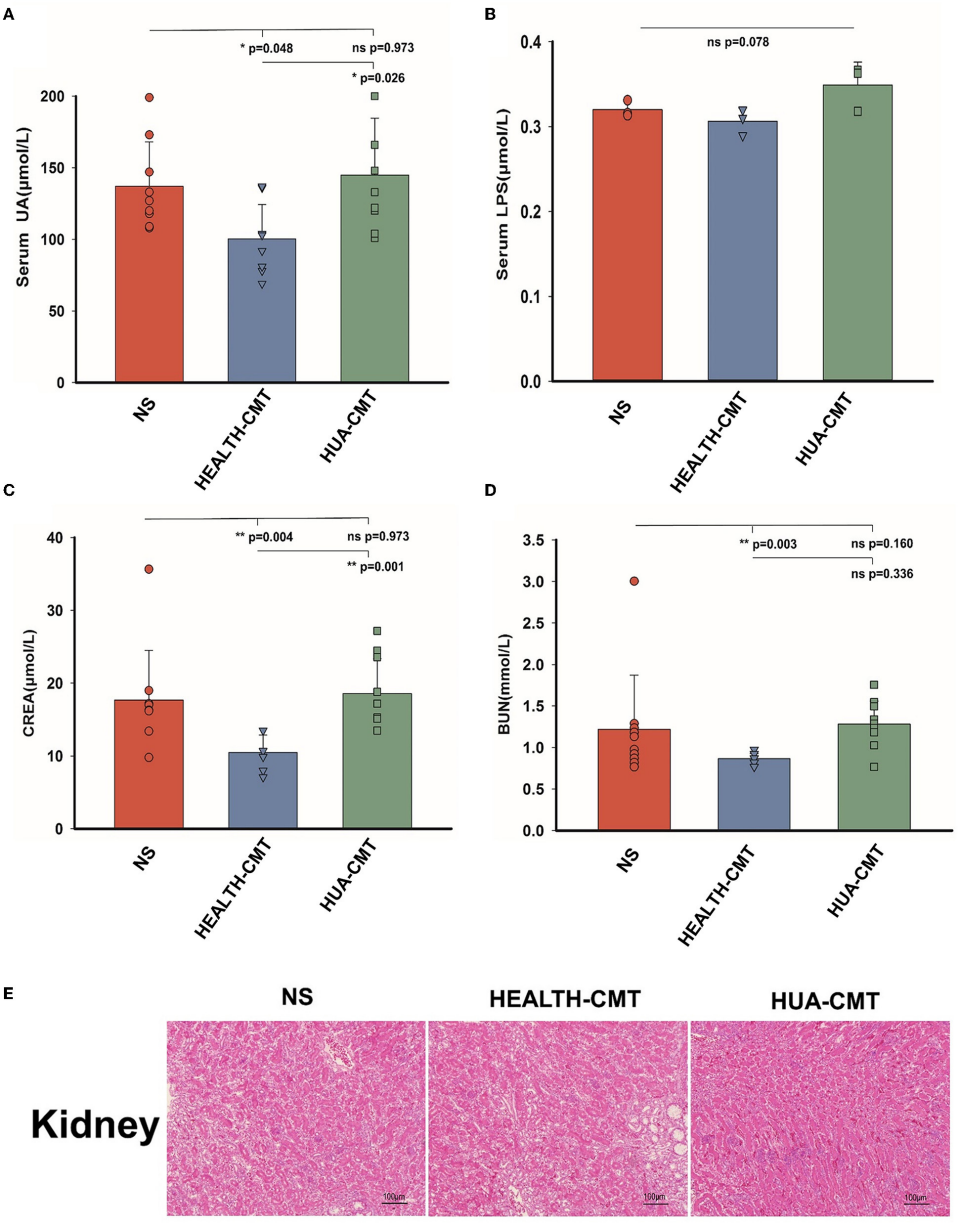
Biochemical indices and Histomorphology of liver and kidney results of goslings after cecal microflora transplantation. **(A)** The serum uric acid levels of the goslings of the four groups. **(B)** The serum LPS levels of the goslings of the four groups. **(C)** The serum creatinine levels of the goslings of the four groups. **(D)** The blood urea nitrogen levels of the goslings of the four groups. **(E)** Representative photomicrographs ( × 100) of HandE staining gosling's liver and kidney tissue. Values are presented as the mean ± SEM. Differences were assessed by One Way ANOVA by the Holm-Sidak method and denoted as follows: *p < 0.05, **p < 0.01, and ***p < 0.001.

Based on the above experiments, we transplanted the cecal microbiota to the newly born goslings with a normal protein diet. Following 25 days of the experiment, there were no discernible changes between the goslings given the HPD group's cecal microbiota and those given the goslings treated with a stroke-physiological saline solution serum uric acid. Nevertheless, the serum uric acid levels of the goslings that transplanted the cecal microbiota of the HPD group were significantly higher than the goslings that transplanted the cecal microbiota of the control group (Figure 5A). After the transplanted cecal microbiota, there was no significant difference in serum LPS between the goslings transplanted cecal microbiota (Figure 5B). There was no significant difference between the NS and HUA-CMT groups in serum creatinine (CREA). Yet, the serum creatinine concentration of the HEALTH-CMT group was significantly lower than the HUA-CMT group (Figure 5C). Furthermore, the blood urea nitrogen (BUN) concentration of the NS group was not considered from the other two groups. However, in the HUA-CMT group, it was higher than the HEALTH-CMT group (Figure 5D). Histopathological analysis showed no significant difference in the kidney between the three groups (Figure 5E). These results revealed that cecal microbiota transplant from HPD groups to newly born goslings elevated uric acid levels and did not cause lesions on the kidney.

### Cecal microbiota transplant from HPD groups to the newly born goslings changes uric acid metabolism

**Figure 6:**
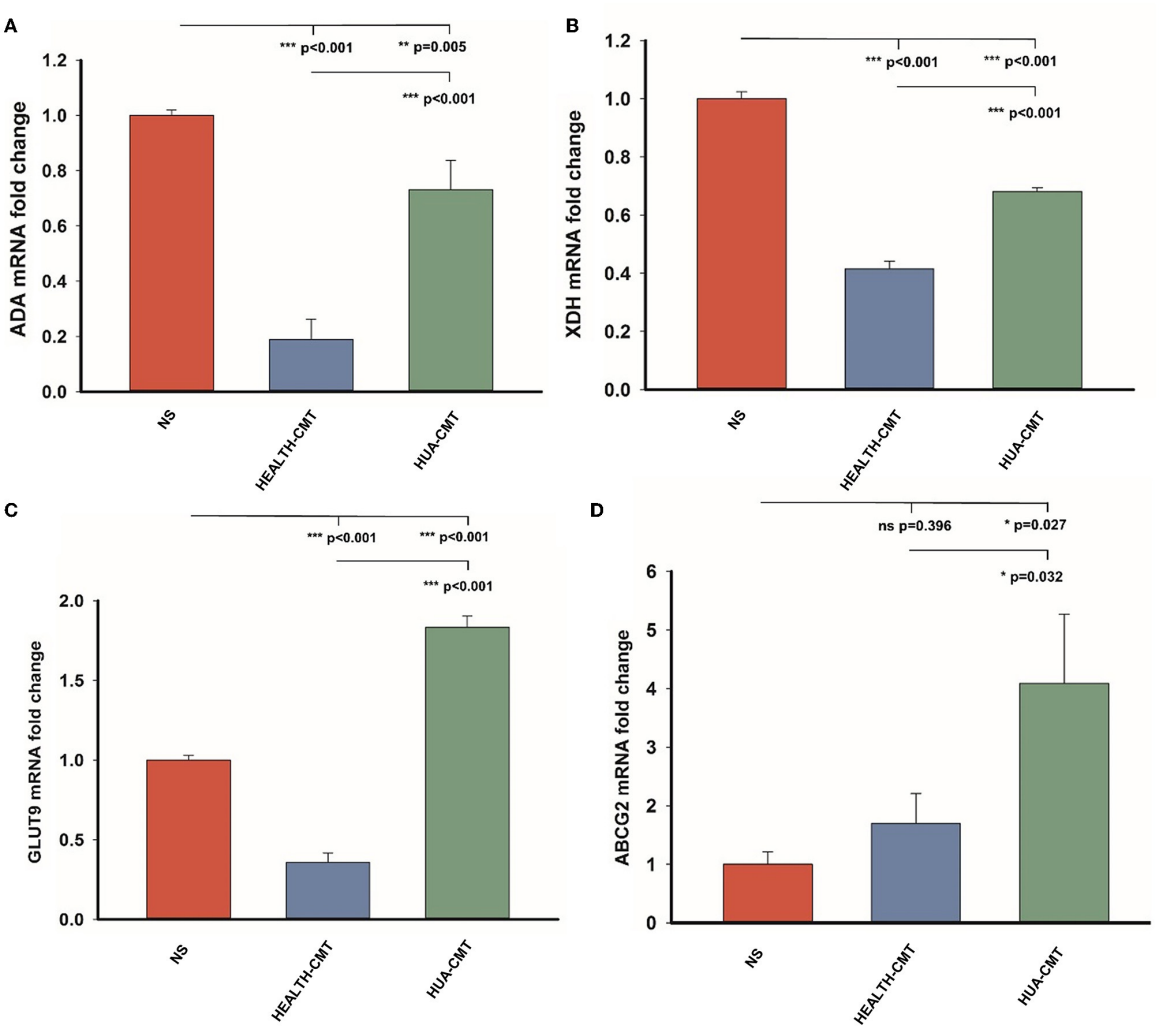
Gene expression of purine metabolic pathway and uric acid transporter after cecal microflora transplantation. **(A)** The mRNA expression level of ADA in the four groups. **(B)** The mRNA expression level of XDH in the four groups. **(C)** The mRNA expression level of GLUT9 in the four groups. **(D)** The mRNA expression level of ABCG2 in the four groups. Values are presented as the mean ± SEM. Differences were assessed by One Way ANOVA by the Holm-Sidak method and denoted as follows: *p < 0.05, **p < 0.01, and ***p < 0.001.

We measured the mRNA expression levels of adenosine deaminase (ADA) and xanthine dehydrogenase (XDH), which affect uric acid synthesis in the liver. We also tested the mRNA expression levels of GLUT9 (SLC2A9) and ABCG2 in the kidney. The GLUT9 is on the kidney's apical surface, which mediates urate transport from the lumen into the cell. ABCG2 is a vital secretory transporter associated with uric acid extraction. In our results, the expression level of ADA in the transplanted cecal microbiota groups was lower than in the NS group. However, the mRNA expression level of ADA in the HUA-CMT group was higher than in the HEALTH-CMT group (Figure 6A). The result of the mRNA expression of the XDH level was very similar to the mRNA expression of ADA (Figure 6B). Meanwhile, the mRNA expression levels of GLUT9 and ABCG2 in the kidney cortex tissue in the HUA-CMT group were higher than in other groups. And the mRNA expression level of ABCG2 in the HEALTH-CMT group was also higher than in the NS group. However, the mRNA expression level of GLUT9 was lower than that in the NS group (Figures 6C, D). These results revealed that cecal microbiota transplant from the HPD group influenced uric acid metabolism, particularly the excretion of uric acid.

### Identification of the isolated strain

**Figure 7:**
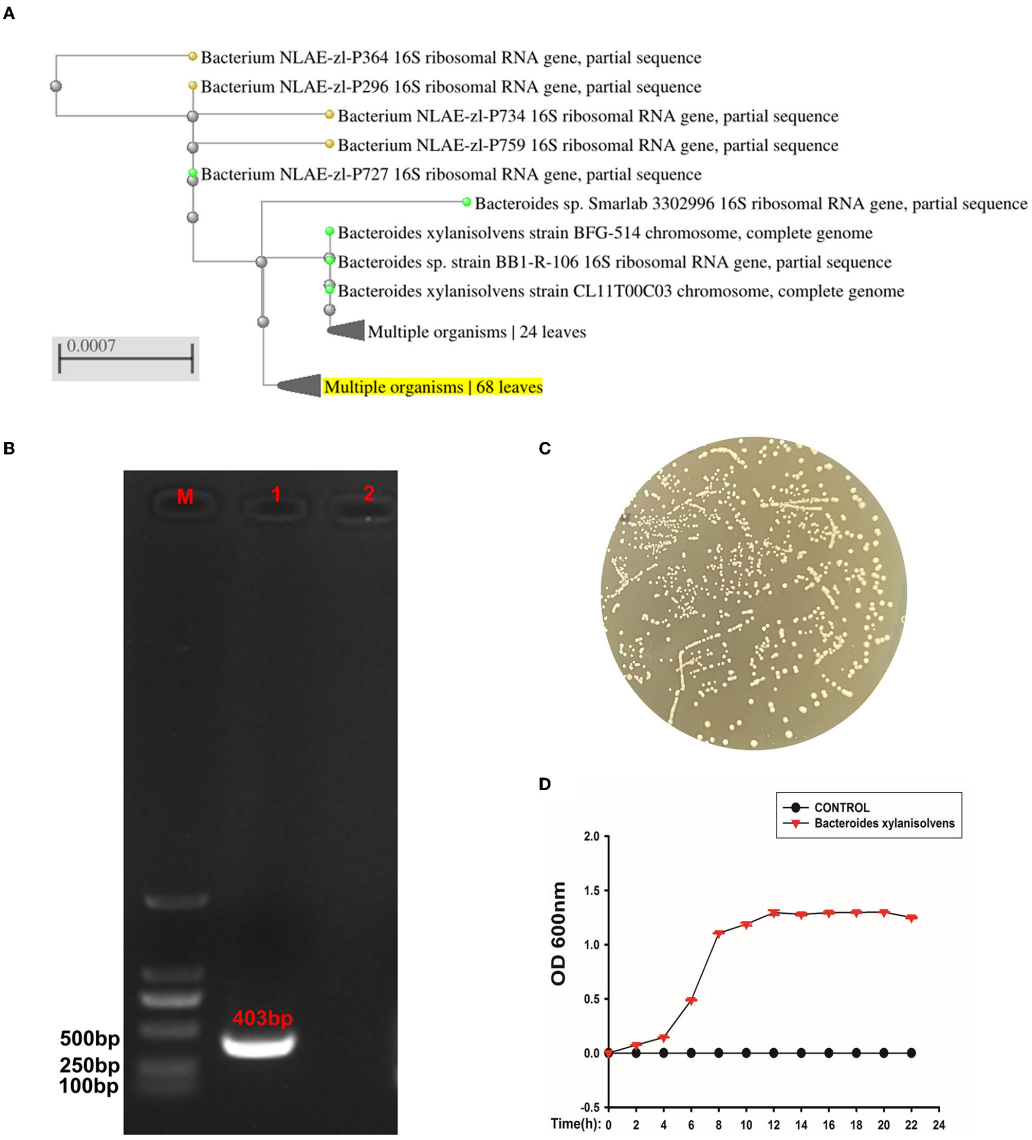
The isolated strain was identified as *Bacteroides xylanisolvens*. **(A)** Phylogenetic tree of strain *Bacteroides xylanisolvens* and related bacteria. **(B)** M is expressed as DNA maker, 1 is expressed as strain *Bacteroides xylanisolvens*, and 2 is expressed as the negative control. **(C)** Colony morphology of *Bacteroides xylanisolvens*. **(D)** Growth curve of *Bacteroides xylanisolvens*.

The isolated strain had a close relationship to *Bacteroides xylanisolvens* compared to the NCBI BLAST database (Figure 7A). The PCR confirmed that the strain was *Bacteroides xylanisolvens*, and the PCR product was 403 bp (Figure 7B). After confirming that the isolates were *Bacteroides xylanisolvens*, we cultured them in BHI solid medium, and the colony morphology is shown in Figure 7C. We then measured its growth curve, which reached a plateau at 12 h (Figure 7D).

### *Bacteroides xylanisolvens* prevent hyperuricemia in goslings caused by high protein diet

**Figure 8:**
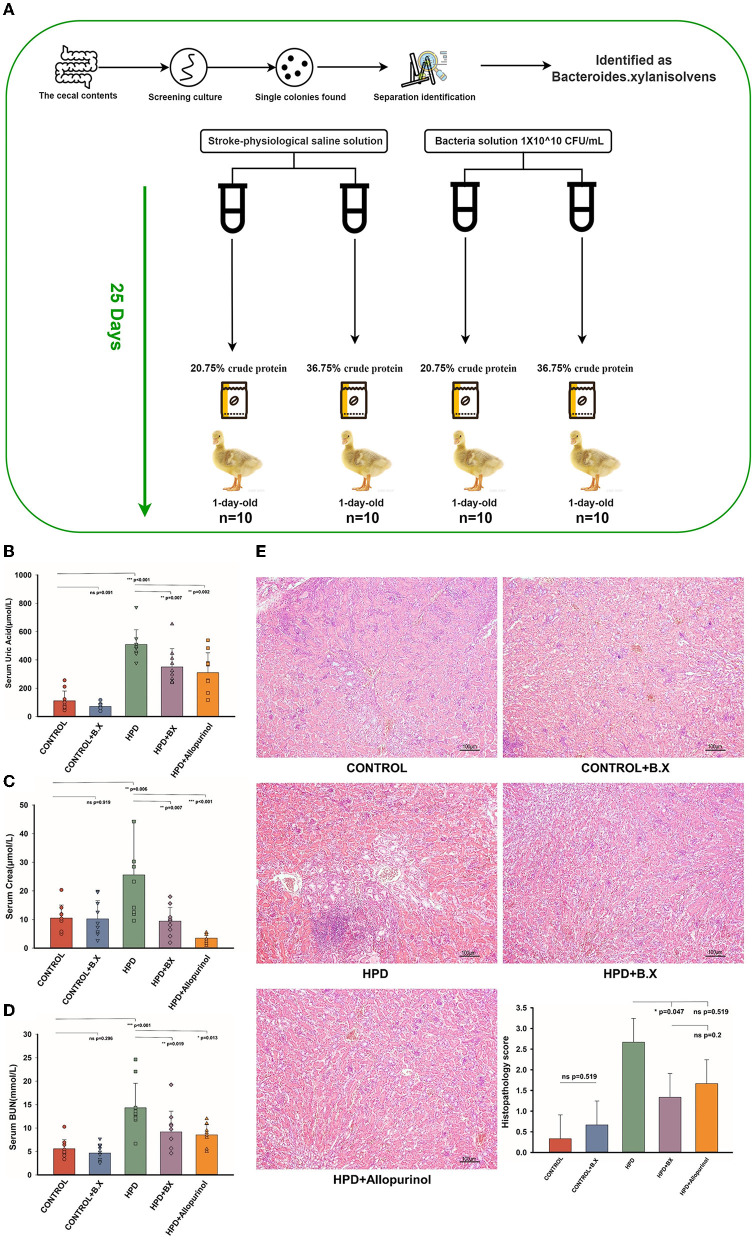
Biochemical indices and Histomorphology of liver and kidney results of goslings after gavaging *Bacteroides xylanisolvens*. **(A)** The experimental process. **(B)** The serum uric acid levels of the goslings of the five groups. **(C)** The serum creatinine levels of the goslings of the five groups. **(D)** The blood urea nitrogen levels of the goslings of the five groups. **(E)** Representative photomicrographs ( × 100) of HandE staining gosling's liver and kidney tissue and histopathology score. Values are presented as the mean ± SEM. Differences were assessed by One Way ANOVA by Tukey test and denoted as follows: *p < 0.05, **p < 0.01, and ***p < 0.001.

HPD or a normal protein diet was fed to goslings for 25 days, and serum uric acid levels were measured. The experiment process is shown in Figure 8A. Serum uric acid levels were higher in the HPD groups than in the other groups. Whether on a normal or high protein diet, the *Bacteroides xylanisolvens* played a role in reducing serum uric acid (Figure 8B). The serum CREA and BUN concentrations of the HPD group were also higher than other groups. While on a high protein diet, the *Bacteroides xylanisolvens* reduced the increase of serum CREA and BUN concentration (Figures 8C, D). Histopathological analysis showed no significant difference in the kidney between the CONTROL and CONTROL+B.X groups (Figure 8E). We found that under a high protein diet, the pathological kidney injury of goslings supplemented with *Bacteroides xylanisolvens* was reduced (Figure 8E).

### *Bacteroides xylanisolvens* change uric acid metabolism in goslings

**Figure 9:**
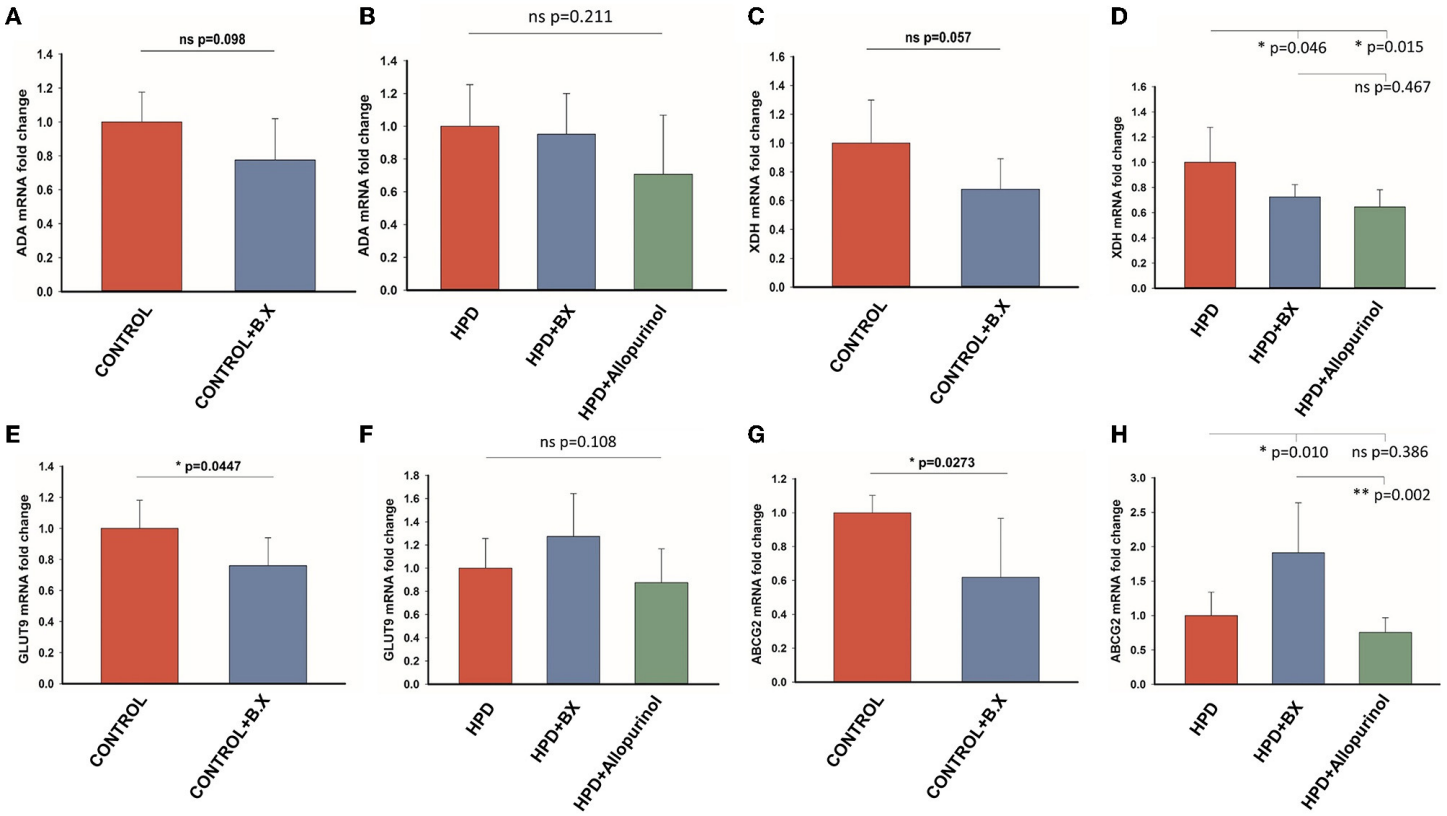
Gene expression of the purine metabolic pathway and uric acid transporter after gavaging *Bacteroides xylanisolvens*. **(A)** The mRNA expression level of ADA in the normal protein diet groups. **(B)** The mRNA expression level of ADA in the high protein diet groups. **(C)** The mRNA expression level of XDH in the normal protein diet groups. **(D)** The mRNA expression level of XDH in the high protein diet groups. **(E)** The mRNA expression level of GLUT9 in the normal protein diet groups. **(F)** The mRNA expression level of GLUT9 in the high protein diet groups. **(G)** The mRNA expression level of ABCG2 in the normal protein diet groups. **(H)** The mRNA expression level of ABCG2 in the high protein diet groups. Values are presented as the mean ± SEM. Differences were assessed by t-test or One Way ANOVA by the Holm-Sidak method and denoted as follows: *p < 0.05, **p < 0.01, and ***p < 0.001.

The effects of *Bacteroides xylanisolvens* on the uric acid metabolism of goslings were investigated in a normal diet and a high protein diet. The expression level of ADA in the CONTROL and CONTROL+B.X groups had no significant differences (Figure 9A), and there was no significant difference in any of the HPD, HPD+B.X, and HPD+Allopurinol groups (Figure 9B). The mRNA expression of the XDH level of the CONTROL and CONTROL+B.X groups showed a significant difference (Figure 9C). Meanwhile, the mRNA expression of the XDH level of the HPD and HPD+B.X groups was also significantly different (Figure 9D). It showed that *Bacteroides xylanisolvens* could down-regulate the mRNA expression of XDH level. In the CONTROL+B.X group, the mRNA expression level of GLUT9 was significantly down-regulated (Figure 9E). But there was no significant difference in the mRNA expression level of GLUT9 in any of the HPD, HPD+B.X, and HPD+Allopurinol groups (Figure 9F). The ABCG2 in the kidney cortex tissue in the CONTROL+B.X group was lower than in the CONTROL group (Figure 9G). However, the mRNA expression level of ABCG2 in the HPD+B.X group was higher than in the HPD group and HPD+Allopurinol (Figure 9H). Our results indicate that *Bacteroides xylanisolvens* can change uric acid metabolism in goslings and reduce the serum uric acid level.

## Discussion

This study used a high-protein diet to induce hyperuricemia in goslings. After a 25-day experiment, it was found that goslings fed with a high-protein diet developed hyperuricemia. Previous reports indicate that poultry can develop severe hypouricemia and gout when fed a high-protein diet (Hong et al., 2020). Since dietary elements that evade host digestion and absorption act as the substrates for intestinal bacteria growth, the diet has the greatest potential to affect the intestinal micro-biome in poultry. Moreover, the cecal microbiota of the HPD group proceeded with 16S rDNA sequencing along with the control group. We found that the gut microbiota had significant changes when hyperuricemia occurred. The control group's abundance of *Bacteroidetes* was higher than that of the HPD group. And the abundance of *Firmicutes* in the HPD group was higher than in the control group. *Poultry Science* also showed the same phenomenon in healthy and gout goslings, which was coincident with our results (Xi et al., 2019). As the LEfSe analysis showed, the Bacteroides may play a vital role in decreasing uric acid and stemming hyperuricemia and gout. Some beneficial effects of Bacteroides have been reported in the literature, such as anti-obesity effects, reduced gut microbial LPS production, and inhibition of atherosclerosis (Yoshida et al., 2018; Lopez-Almela et al., 2021). Moreover, under the normal protein diet, the 1-day-old goslings were transplanted with the cecal microbiota of the control group, and their serum uric acid level was lower than other groups. The serum uric acid levels of the goslings that transplanted the cecal microbiota of the HPD group were higher than the goslings that transplanted the cecal microbiota of the NS group, although there was no significant difference between these two groups. This result further shows that intestinal flora plays an essential role in the occurrence and development of hyperuricemia. It also shows that the more healthy intestinal flora is, the less likely the body will develop hyperuricemia, as it has a curbing effect on the development of hyperuricemia.

The imbalance between UA production and excretion is considered to result in hyperuricemia (Johnson et al., 2018). Furthermore, hyperuricemia is also associated with elevated purine degradation, metabolic acidosis, and renal insufficiency (Pilemann-Lyberg et al., 2019; Jung et al., 2020). Current studies show intestinal flora is closely related to hyperuricemia and gout and that gut microbiota affects the production, secretion, and metabolism of uric acid in the gut (Wang et al., 2022). Under normal circumstances, cecal microbial flora normally fluctuates within a certain range of species and quantities. Numbers of harmful bacteria multiply when there is a disease or stress present. Furthermore, hyperuricemia and gout have been linked to the microbiome and metabolome of the feces (Chu et al., 2021). While the leading production site of uric acid is in the liver, the leading site of uric acid excretion in humans and birds is the kidney.

We found that the expression of ADA and XDH in the goslings transplanted cecal microbiota of the HUA group was higher than in the control group. At the same time, we also found that the expression of GLUT9 and ABCG2 in the transplanted cecal microbiota of the HPD group goslings was higher than that of the goslings transplanted cecal microbiota of the control group. Both ADA and XDH affected uric acid synthesis in the liver, suggesting intestinal microbiota may also affect uric acid synthesis. Furthermore, GLUT9 dominated the reabsorption of uric acid in the kidney, and ABCG2 dominated the excretion of uric acid, which indicates that intestinal flora may also affect the excretion of uric acid. The high expression of renal transporters also demonstrated that geese have a compensatory mechanism for protection from external damage (Ma et al., 2021). Furthermore, the experimental results are also in line with the prediction of TaX4FUN for the function of intestinal flora. This result further indicates that intestinal flora may affect the overall metabolism of uric acid. We can speculate that it is due to the uric acid-lowering effect of certain bacteria in the healthy flora. On the other hand, it may be that certain harmful bacteria dominate the damaged intestinal flora, resulting in increased uric acid levels in the body, further aggravating the occurrence of hyperuricemia and gout. According to the above results, we did further work and isolated a strain of *Bacteroides xylanisolvens* by the specific medium of *Bacteroides*. The *B. xylanisolvens*, first isolated from human feces as a novel *Bacteroides* species, possessed a high xylanase activity with the type strain named XB1A (Chassard et al., 2008). A few studies have reported that *B. xylanisolvens* DSM 23964 increases the concentration of TFa-specific IgM serum antibodies that are involved in controlling cancer development (Schmidt et al., 2016) and exhibit no virulence in humans (Ulsemer et al., 2012). We found that our newly isolated *Bacteroides xylanisolvens* could inhibit hyperuricemia in goslings by up-regulating ABCG2 mRNA expression in the kidney and down-regulating XDH mRNA expression in the liver.

The primary purpose of this experiment was to study the relationship between intestinal flora and hyperuricemia in goslings. The results showed a unity of opposites relationship between gut microbiota and hyperuricemia, with alterations in gut microbiota due to high uric acid in the blood. This change, in turn, can further exacerbate hyperuricemia. Our findings suggested that gut microbiota may influence uric acid synthesis in the liver and excretion in the kidneys, and we explored the relationship between intestinal flora and hyperuricemia. As a result of our findings, we may be able to provide insight into the prevention of gout and hyperuricemia in poultry as well as a dietary therapy for mammals. Moreover, it provided a research basis for treating hyperuricemia from the perspective of intestinal flora in the future. *Bacteroides xylanisolvens* may play an important role in gout and hyperuricemia in goslings.

## Conclusion

Hyperuricemia is a prevalent physiological component contributing to gout in humans and birds. In our experiment, we investigated how intestinal flora affects gosling hyperuricemia. We discovered that transplanted cecal microbiota could have an impact on goslings' serum uric acid levels, as well as their uric acid production and excretion. We found that *Bacteroides xylanisolvens* work well to prevent hyperuricemia in goslings. Our findings may help in the development of nutritional therapy for mammals as well as the prevention of gout and hyperuricemia in poultry. *Bacteroides xylanisolvens* may play a role in treating gout and hyperuricemia in goslings.

## Data availability statement

The datasets presented in this study can be found in online repositories. The names of the repository/repositories and accession number(s) can be found below: http://db.cngb.org/cnsa/project/CNP0002797_715a6b1c/reviewlink/, CNP0002797.

## Ethics statement

The animal study was reviewed and approved by Institutional Animal Care and Use Committee of Jilin University.

## Author contributions

NZ and XL conceptualized the study. NS was responsible for software applications, investigation, and writing the original draft. KZ and MW helped with validation and analysis. NS and GZ were responsible for resources and data curation. WZ and PC wrote and revised the final draft of the manuscript. All authors read and approved the published version of the manuscript.
