# Supplementary material for: Bacteroides xylanisolvens possesses a potent anti-hyperuricemia effect in goslings fed on a high-protein diet
Source: Front Microbiol. 2023 Jun 30;14:1173856. doi: 10.3389/fmicb.2023.1173856 (PMC10348916; doi:10.3389/fmicb.2023.1173856)

**Supplementary materials**

**The sensitivity and specificity of the primer**

**Sensitivity detection**

When the number of well-cultivated *Bacteroides xylanisolvens* reached 2 × 10^10^ cells/ml, and diluted to 10^9^ cells/ml, then extracted DNA. Ten microliters of the extracted DNA solution were pipetted into 90 μL of TE to prepare *Bacteroides xylanisolvens* DNA solution of 10^8^ and then diluted to 10^0^ in a 10-fold gradient.

The results showed that the sensitivity of the primer for PCR was high, and the DNA content of one *Bacteroides xylanisolvens* was detected (S1 Fig7A).

**Specificity test**

We obtained seven kinds of bacteria from College of Veterinary Medicine, Jilin University, then extracted DNA for detection.

*Leptospira strain Lai 56601, Staphylococcus aureus, Bacillus subtills, Lactobacillus*, *Escherichi coli*, *Legionella, Enterococcus faecalis,* negative control has no specific bands, indicating specificity was good (S1 Fig7B). The sequence comparative analysis result shows it was *Bacteroides xylanisolvens*. The amplification sequences were showed in S1Table1.

***
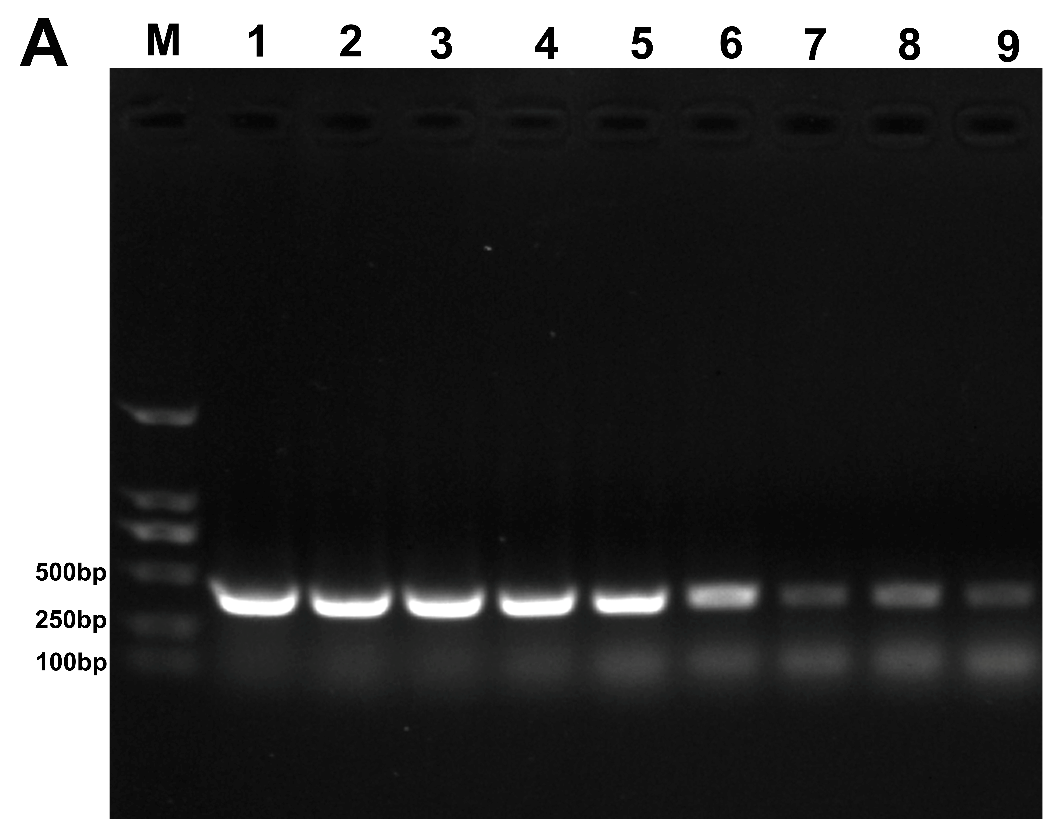
***

**S1: Fig7A: The sensitivity of the primers for PCR. M is the DNA marker, and 1-9 indicates the concentration gradient of** ***Bacteroides xylanisolvens*: 10 ^8^ -10 ^0^.**

***
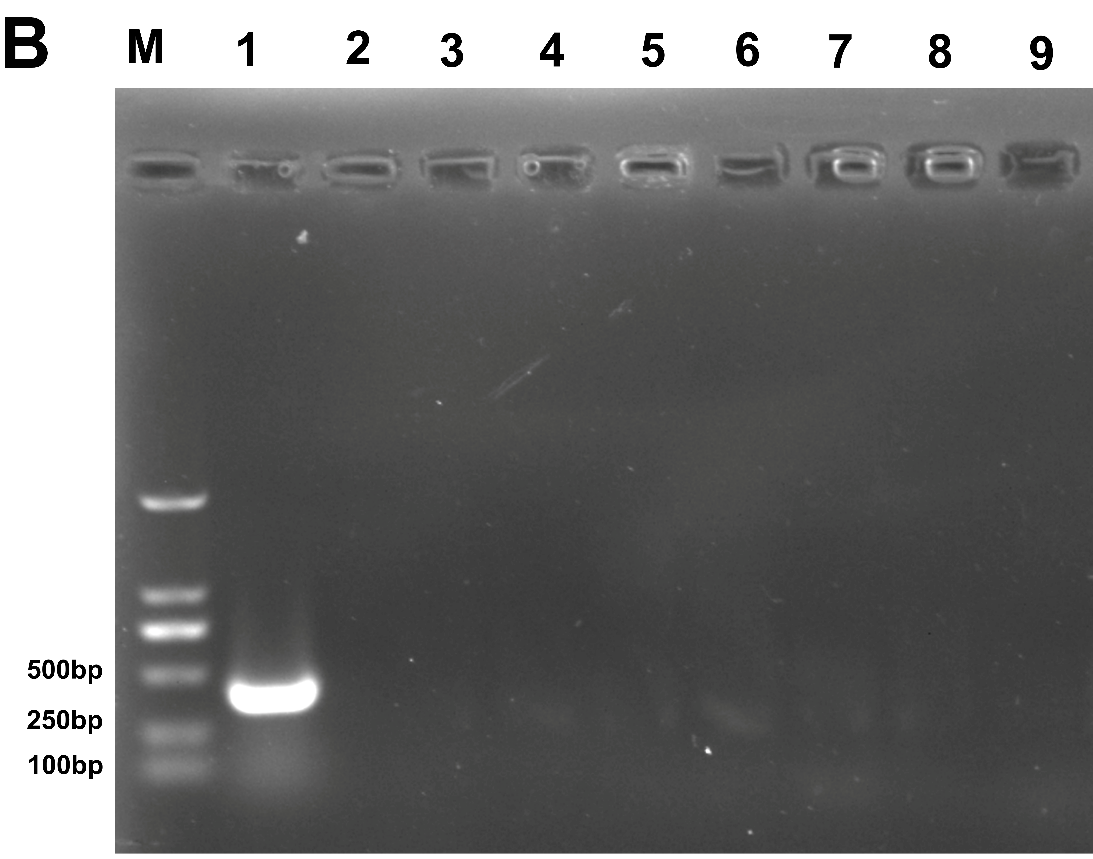
***

**Fig7B: M is expressed as DNA maker, 1-9 is expressed as:** ***Bacteroides xylanisolvens, Leptospira strain Lai 56601, Staphylococcus aureus, Bacillus subtills, Lactobacillus*, *Escherichi coli*, *Legionella, Enterococcus faecalis,* negative control.**

**S1: Fig7A:** The sensitivity of the primers for PCR. M is the DNA marker, and 1-9 indicates the concentration gradient of *Bacteroides xylanisolvens*: 10 ^8^ -10 ^0^. Fig7**B:** M is expressed as DNA maker, 1-9 is expressed as: *Bacteroides xylanisolvens, Leptospira strain Lai 56601, Staphylococcus aureus, Bacillus subtills, Lactobacillus, Escherichi coli, Legionella, Enterococcus faecalis, negative control.*

S1 Table1 The newly isolated *Bacteroides xylanisolvens* PCR production amplification sequences(373bp)

| TGAGAAGAACCTTTATGATGAAAGCAAGCAACCTGAAAAAGAAATTCAAGTAAAAGACCTGGATATACCCGCAGGCTTTCAATGGAAATTAACTCAAGTAGCCGCAGGAACAGTAGCTGCTACTACCCCCACTATGGTTTCCTTCTTTTTGGATGAAGCATGTAGTAAAGAAGAGAAAATAGCCGACATTCCTGTAGATACAGAAATTTCAAGTCTCCCTTTGAGCATCCCAACCTACGTAAATACATTGTATGCCCAGTACAAAACCAGCACCAATGAAACCAAAAAAGTAGCCATACCTGTAAATGCAGATAGAAGTTTCTCACTGAACATTGCTAATGATGCCAAATCCAAATCTAACACCACCCGGTCA |
| --- |

**The 16S sequencing and gut microbiota analysis quality control**

**Species diversity curve**

Rarefaction curve and Rank abundance curve are common curves to describe the diversity of samples within a group. The dilution curve is to randomly select a certain amount of sequencing data from the sample, count the number of species they represent (i.e., the number of OTUs), and construct the curve based on the amount of sequencing data extracted and the corresponding number of species. The dilution curve can directly reflect the rationality of sequencing data volume and indirectly reflect the richness of species in the sample. When the curve tends to be flat, it indicates that the sequencing data volume is gradually reasonable, and more data volume will only produce a small number of new species (OTUs).

The hierarchical clustering curve is to sort the OTUs in the sample according to the relative abundance (or the number of sequences contained) from large to small to obtain the corresponding sorting number, and then take the sorting number of OTUs as the abscissa, the relative abundance in OTUs (or the relative percentage content of the number of sequences in the OTUs of this grade) as the ordinate, and connect these points with broken lines to draw the Rank Abundance curve, which can directly reflect the richness and uniformity of species in the sample. In the horizontal direction, the richness of species is reflected by the width of the curve. The higher the richness of species, the greater the span of the curve on the horizontal axis. In the vertical direction, the smoothness of the curve reflects the uniformity of species in the sample. The flatter the curve, the more uniform the distribution of species. In the dilution curve, the abscissa is the number of sequencing strips randomly selected from a sample, and the ordinate is the number of OTUs that can be constructed based on the number of sequencing strips, which are used to reflect the sequencing depth. Different samples are represented by curves with different colors;In the Rank Abundance curve, the abscissa is the sequence number sorted by OTUs abundance, the ordinate is the relative abundance of corresponding OTUs, and different samples are represented by broken
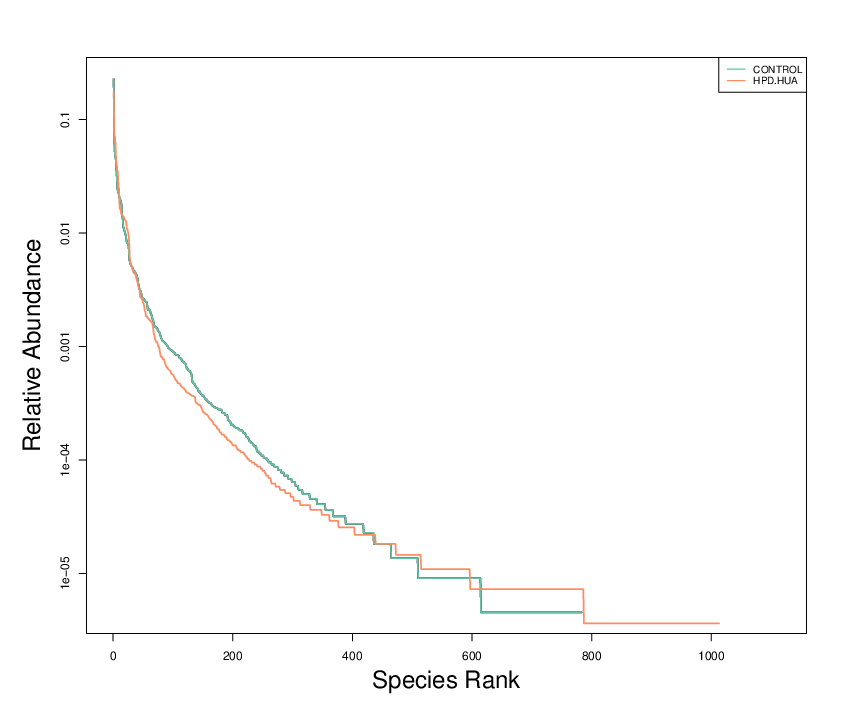
lines with different colors


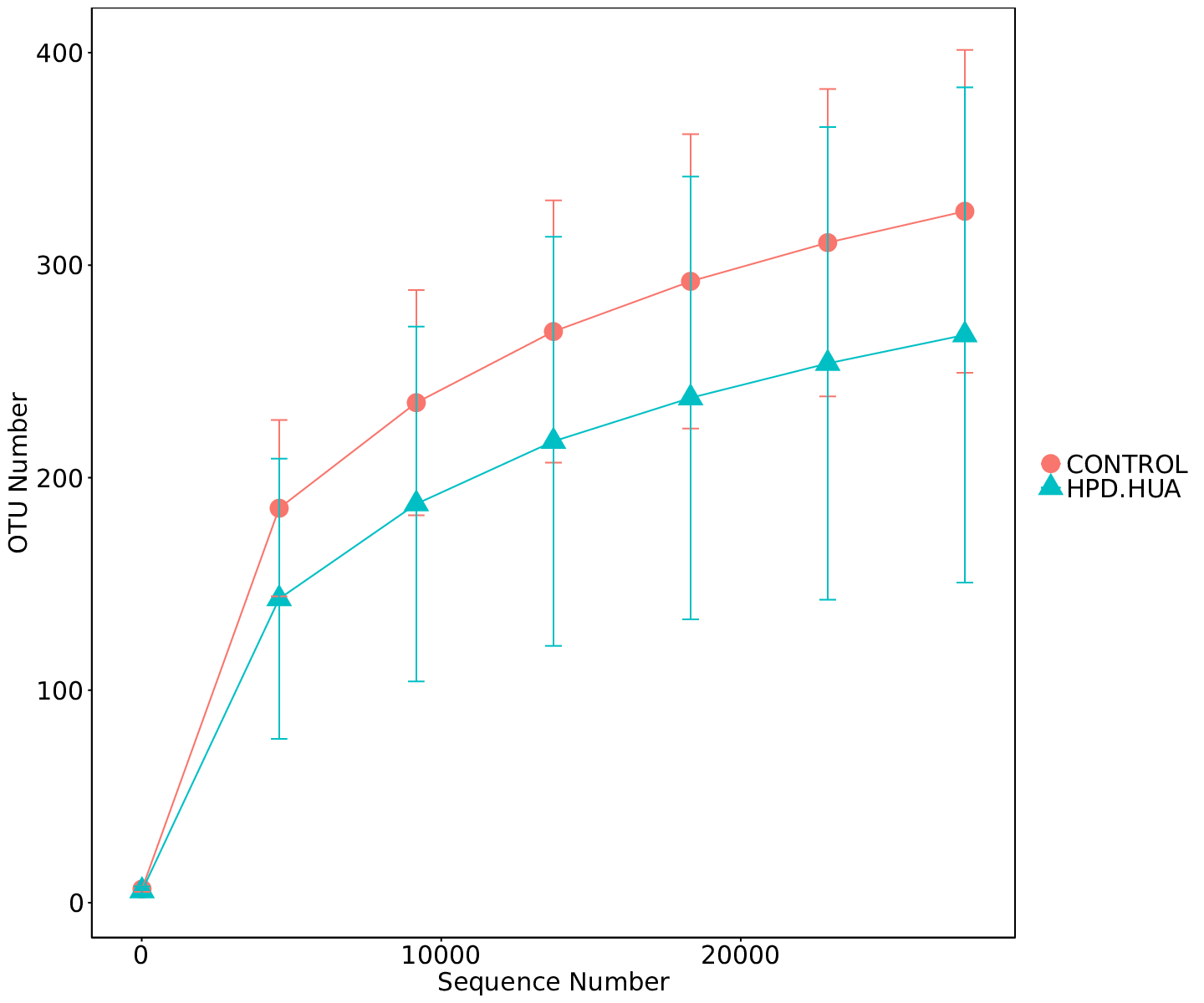


**Species accumulation boxplot**

Species accumulation boxplot is an analysis describing the increase of species diversity with the increase of sample size. It is an effective tool for investigating species composition of samples and predicting species abundance in samples. It is widely used to judge whether sample size is sufficient and estimate species richness in biodiversity and community investigation. Therefore, we can not only judge whether the sample size is sufficient or not, but also predict the species richness by using the species cumulative box plot when the sample size is sufficient. The abscissa is the sample size; the ordinate is the number of OTUs after sampling. The results reflect the rate at which new OTUs (new species) appear under continuous sampling. In a certain range, with the increase of sample size, if the box plot position showed a sharp rise, it means that a large number of species were found in the community;When the boxplots flatten out, it means that the species in the environment do not increase significantly with the increase in sample size. The boxplot of species accumulation can be used to judge whether the sample size is sufficient or not. The sharp rise of boxplot position indicates that the sample size is insufficient and needs to be increased.if not, that sample is sufficient for data analysis.


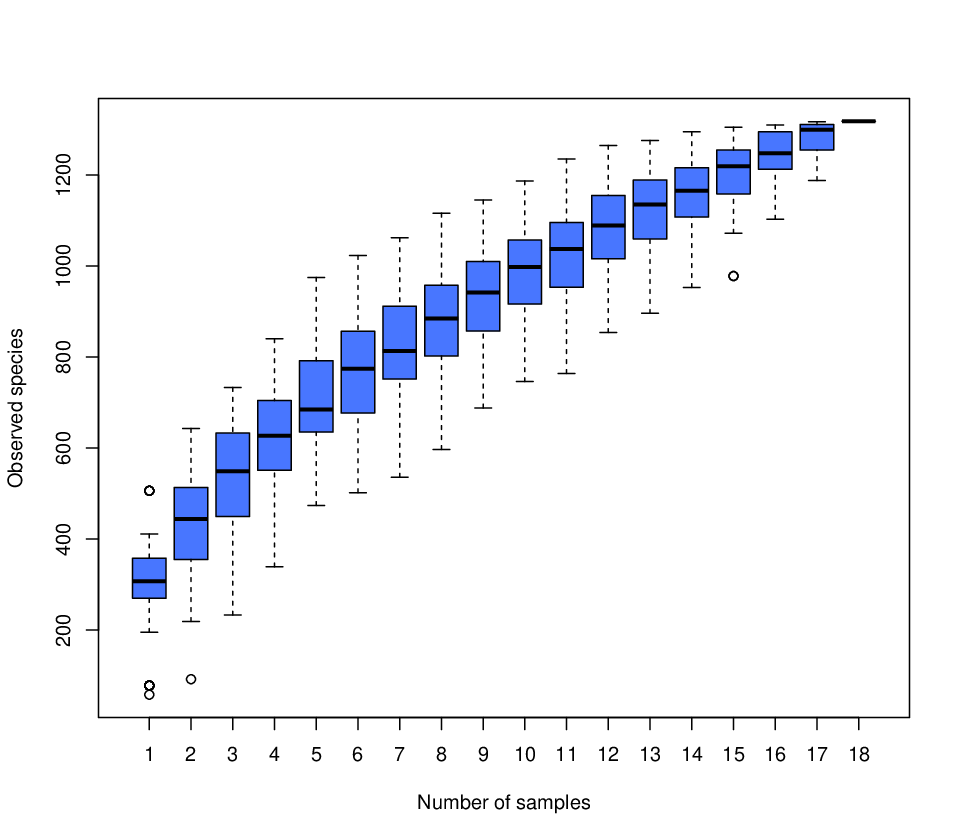

Supplement: Supplementary file 1 [file Data_Sheet_1.DOCX]
